# Step Count, Self-reported Physical Activity, and Predicted 5-Year Risk of Atrial Fibrillation: Cross-sectional Analysis

**DOI:** 10.2196/43123

**Published:** 2023-03-06

**Authors:** Ayelet Shapira-Daniels, Jelena Kornej, Nicole L Spartano, Xuzhi Wang, Yuankai Zhang, Chathurangi H Pathiravasan, Chunyu Liu, Ludovic Trinquart, Belinda Borrelli, David D McManus, Joanne M Murabito, Emelia J Benjamin, Honghuang Lin

**Affiliations:** 1 Section of General Internal Medicine, Department of Medicine Boston Medical Center Boston University Chobanian and Avedisian School of Medicine Boston, MA United States; 2 Boston University's Framingham Heart Study National Heart, Lung, and Blood Institute Framingham, MA United States; 3 Section of Endocrinology, Diabetes, Nutrition and Weight Management, Department of Medicine Boston Medical Center Boston University Chobanian and Avedisian School of Medicine Boston, MA United States; 4 Department of Biostatistics Boston University School of Public Health Boston, MA United States; 5 Center for Behavioral Science Research Henry M Goldman School of Dental Medicine Boston University Boston, MA United States; 6 Department of Medicine University of Massachusetts Chan Medical School Worcester, MA United States; 7 Department of Quantitative Health Sciences University of Massachusetts Chan Medical School Worcester, MA United States; 8 Department of Epidemiology Boston University School of Public Health Boston, MA United States; 9 Section of Cardiovascular Medicine, Department of Medicine Boston Medical Center Boston University Chobanian and Avedisian School of Medicine Boston, MA United States

**Keywords:** atrial fibrillation, physical activity, fitness tracker, cardiovascular epidemiology, fitness, exercise, tracker, cardiology, heart, walk, step count, smartwatch, wearable, risk, cross-sectional analysis

## Abstract

**Background:**

Physical inactivity is a known risk factor for atrial fibrillation (AF). Wearable devices, such as smartwatches, present an opportunity to investigate the relation between daily step count and AF risk.

**Objective:**

The objective of this study was to investigate the association between daily step count and the predicted 5-year risk of AF.

**Methods:**

Participants from the electronic Framingham Heart Study used an Apple smartwatch. Individuals with diagnosed AF were excluded. Daily step count, watch wear time (hours and days), and self-reported physical activity data were collected. Individuals’ 5-year risk of AF was estimated, using the Cohorts for Heart and Aging Research in Genomic Epidemiology (CHARGE)–AF score. The relation between daily step count and predicted 5-year AF risk was examined via linear regression, adjusting for age, sex, and wear time. Secondary analyses examined effect modification by sex and obesity (BMI≥30 kg/m^2^), as well as the relation between self-reported physical activity and predicted 5-year AF risk.

**Results:**

We examined 923 electronic Framingham Heart Study participants (age: mean 53, SD 9 years; female: n=563, 61%) who had a median daily step count of 7227 (IQR 5699-8970). Most participants (n=823, 89.2%) had a <2.5% CHARGE-AF risk. Every 1000 steps were associated with a 0.08% lower CHARGE-AF risk (*P*<.001). A stronger association was observed in men and individuals with obesity. In contrast, self-reported physical activity was not associated with CHARGE-AF risk.

**Conclusions:**

Higher daily step counts were associated with a lower predicted 5-year risk of AF, and this relation was stronger in men and participants with obesity. The utility of a wearable daily step counter for AF risk reduction merits further investigation.

## Introduction

Atrial fibrillation (AF) is the most common cardiac arrhythmia, and it is an important cause of stroke, heart failure, and death [1]. The Cohorts for Heart and Aging Research in Genomic Epidemiology (CHARGE)–AF score is a tool that has been validated to estimate an individual’s 5-year risk of developing AF, using relevant clinical information and known risk factors, such as height, weight, blood pressure, and a history of heart failure and myocardial infarction [2-4].

In recent years, researchers have investigated AF risk modification via lifestyle changes, such as decreased alcohol consumption, weight loss, smoking cessation, and other factors [5-11]. In particular, physical activity has been examined as a means to decrease the risk of cardiovascular disease (CVD) and AF [12-20]. Studies that examine objectively measured physical activity and AF have mostly used research-grade accelerometers or implantable loop recorders, which limit applicability to daily life [16-20]. Although research-grade accelerometers provide superior validity and precision in their ability to reflect an individual’s physical activity, they are not as widely available to the general public as commercially available wearable devices [21]. Additionally, in studies that measured physical activity with research-grade accelerometers, physical activity data were collected for only a brief period of time (4-7 days) [17,18]. The sharp rise in the prevalence of wearable devices with step counters has resulted in a unique opportunity for health management and optimization, with direct applicability to individuals’ lifestyles. To our knowledge, a direct relation between the long-term tracking of daily step counts from commercially available wearable devices and the risk of AF has yet to be investigated.

We hypothesized that a higher daily step count, as measured by wearable devices, is associated with a lower 5-year risk of AF, as predicted by the CHARGE-AF score.

## Methods

### Study Sample

The Framingham Heart Study (FHS), which originated in 1948 to investigate CVD, is a community-based cohort study that spans 3 generations of families [22,23]. In recent years, participants from the Third Generation Cohort, multiethnic Omni Group 2 Cohort, and New Offspring Spouse Cohort were invited to enroll in the electronic FHS (eFHS) at the time of their third research examination (2016-2019) [24]. The use of the Apple Watch (Series 0; Apple Inc)—a smartwatch that allows for the tracking of daily steps and heart rate—was incorporated into the eFHS in 2016. Participants were required to be English speakers and own an iPhone (Apple Inc) to be eligible for the study.

### Ethics Approval

This study was approved by the Institutional Review Board of Boston University Medical Center (approval number: H-36586). All participants provided written informed consent.

### Collection of Objective Physical Activity Data

In this analysis, we selected eFHS participants, as displayed in Figure S1 in Multimedia Appendix 1. Of the 3521 participants examined in the research center, 1948 had a compatible iPhone, provided consent, and were ultimately enrolled in our eFHS sample. Only 1185 used the Apple Watch and returned step data. We excluded 244 participants who did not wear the watch for >30 days and 18 participants with prevalent AF. Participants were encouraged to wear their Apple Watch daily.

The total number of hours of watch wear time per day and the number of days participants wore the watch were recorded. A *wear hour* was defined as an hour with at least one heart rate measure or the time when at least 30 steps were accumulated. An *active day* was defined as a day with at least 5 watch wear hours. *Average daily step count* and *watch wear time* were defined as participants’ mean daily step counts and their total wear hours, respectively, for active days.

### Estimation of AF Risk

The primary dependent measure was participants’ 5-year risk of AF, which was estimated based on the CHARGE-AF risk scores that were calculated by using the clinical risk factors assessed when participants were examined at the FHS research center [24]. This previously validated prediction model is a Cox proportional hazard regression model. The prediction model uses an individual’s age (years), height (cm), weight (kg), self-reported race and ethnicity, systolic and diastolic blood pressure (mm Hg), current smoking status, antihypertensive medication use, history of diabetes mellitus, history of heart failure, and history of myocardial infarction to predict the individual’s 5-year risk of AF.

### Self-reported Physical Activity

Participants were asked to complete a questionnaire during their FHS research examinations to determine their physical activity levels. This questionnaire, which has been used in other FHS studies, asked participants to estimate the number of hours in a typical day that they spent performing varying levels of physical activity over the past year [25,26]. We then calculated a physical activity index (PAI) score as a weighted composite of hours per day spent performing activities of varying physical intensity. For example, sleeping was weighted at 1.0; slight activity, such as standing and walking, was weighted at 1.5; and high-intensity activity, such as jogging and swimming, was weighted at 5. As such, PAI scores could hypothetically range from 24 (eg, 24 hours per day of sleeping) to 120 (eg, 24 hours per day of high-intensity exercise), with higher PAI scores indicating a participant’s perception of higher daily physical activity levels.

### Statistical Methods

Daily step count, watch wear time, and clinical variables were reported as means with SDs for continuous variables and as n values with percentages for dichotomous variables. When continuous variable distribution was skewed, medians with IQRs were reported.

The primary analysis examined the association between daily step count (independent measure) and the CHARGE-AF risk score (dependent measure) via linear regression, adjusting for age, sex, and average wear time per day. Both daily step count and the CHARGE-AF score were treated as continuous variables.

Secondary analyses tested for interactions between daily step count and sex and between daily step count and obesity (BMI≥30 kg/m^2^) in their association with the CHARGE-AF risk score, given that male sex and obesity are established independent risk factors for AF. For graphic purposes, we performed an analysis that examined the CHARGE-AF score, as a dependent variable, in high versus low physical activity groups (<7500 vs ≥7500 daily steps). High and low physical activity groups were determined by using the average step count of the study sample as the cutoff. The analysis was performed by using a Wilcoxon rank-sum test and adjusted for age.

Additional analyses examined the association between self-reported physical activity (PAI score) and CHARGE-AF risk via linear regression, adjusting for age and sex. Sensitivity analyses were performed by using different thresholds for watch wear time (5 vs 10 hours/day) and number of active days (30 vs 60 vs 90 days). A 2-sided *P* value of <.05 was considered statistically significant. All of the analyses were performed by using R software package version 4.0.3 (R Foundation for Statistical Computing).

## Results

### Participant Characteristics

**Figure 1:**
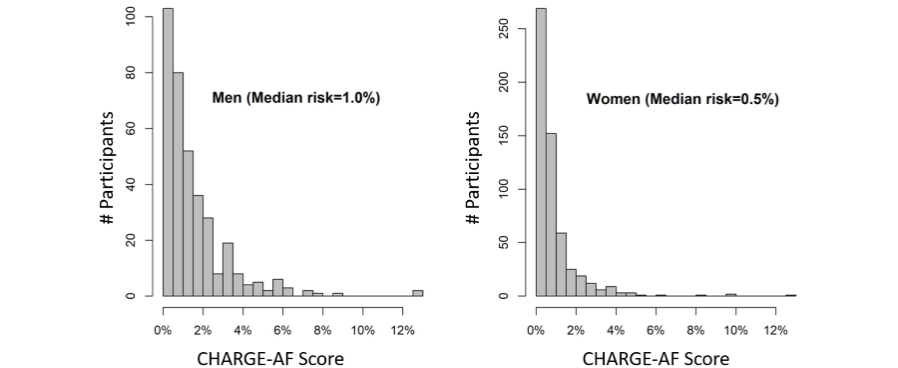
Distribution of 5-year AF risk among participants. Most participants (823/923, 89.2%) had a 5-year AF risk of <2.5%, as determined by the CHARGE-AF score. AF: atrial fibrillation; CHARGE: Cohorts for Heart and Aging Research in Genomic Epidemiology.

We included 923 participants in this study. The mean age of participants was 53 (SD 9) years, 563 (61%) participants were female, and 838 (90.8%) participants identified as White. The median daily step count was 7227 (IQR 5699-8970), and the median watch wear time was 13.6 (IQR 12.4-14.7) hours per day for 324 (IQR 137-563) active days. The median CHARGE-AF risk score was 1% for men and 0.5% for women (Figure 1). Participants’ mean PAI score, which we calculated based on their self-reported physical activity levels, was 33.4 (SD 4.7; range 24-120; lower scores indicate less self-reported physical activity). Additional demographic characteristics can be seen in Table 1.

**Table 1:** Characteristics of the study participants.

Characteristics^a^		Participants (N=923)	
Age (years), mean (SD)		53 (9)	
Sex (female), n (%)		563 (61)	
BMI (kg/m^2^), mean (SD)		28.2 (5.5)	
Height (cm), mean (SD)		169 (9)	
Weight (kg), mean (SD)		81 (18)	
Systolic blood pressure (mm Hg), mean (SD)		118 (14)	
Diastolic blood pressure (mm Hg), mean (SD)		76 (9)	
History of heart failure, n (%)		6 (0.7)	
History of myocardial infarction, n (%)		12 (1.3)	
Current smoking, n (%)		44 (4.8)	
Diabetes mellitus, n (%)		53 (5.7)	
Antihypertensive medication use, n (%)		193 (20.9)	
Self-reported physical activity index score, mean (SD)		33.4 (4.7)	
**Self-reported race and ethnicity, n (%)**			
	White		838 (90.8)
	Black		21 (2.2)
	Asian		17 (1.8)
	Hispanic		27 (2.9)
	Other		20 (2.2)
Daily step count, median (IQR)			7227 (5699-8970)
Active hours per day, mean (SD)			13.4 (1.9)
Active days, median (IQR)			324 (137-562)

^a^Presented are means with SDs for continuous traits with a normal distribution, medians with IQRs for continuous traits with a skewed distribution, and n values with percentages for dichotomous traits.

### Association Between Daily Step Count and 5-Year Risk of AF

After adjusting for age, sex, and watch wear time, our primary analysis, in which linear regression was performed, showed that daily step count and the CHARGE-AF score were inversely associated. Every 1000 steps were associated with a 0.08% lower CHARGE-AF risk score (Table 2).

**Figure 2:**
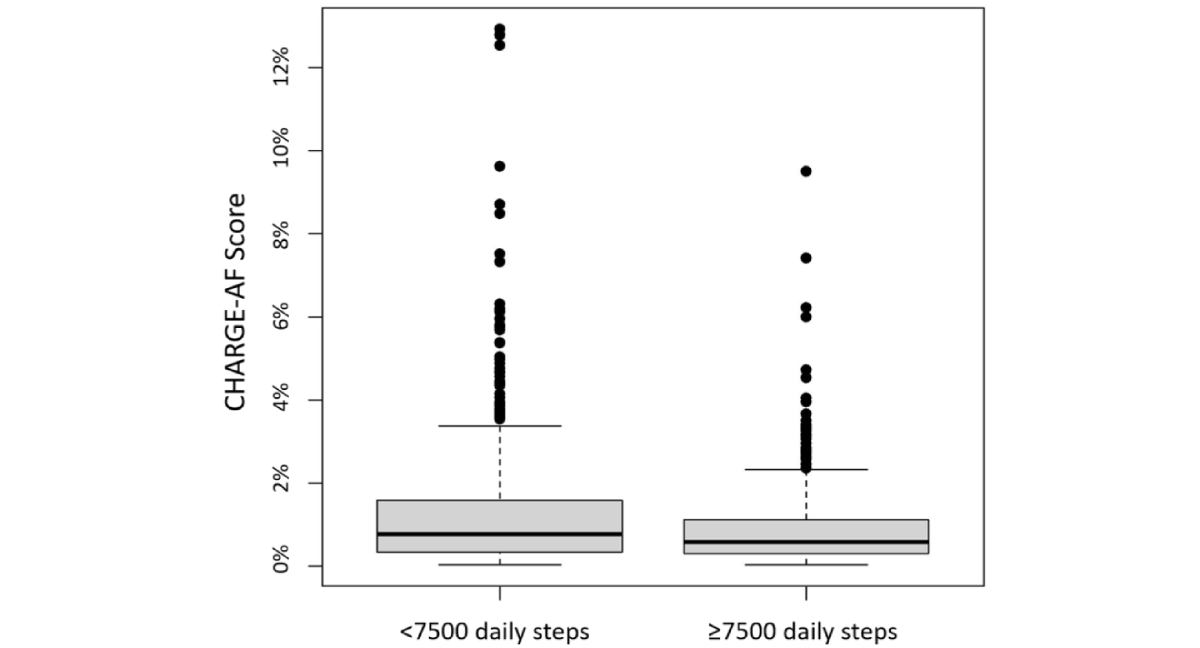
Participants' 5-year AF risk by high versus low daily step count. Participants with lower daily step counts had higher CHARGE-AF scores (mean 1.3% for low daily step count vs mean 0.9% for high daily step count; *P*<.001). The model was adjusted for age. AF: atrial fibrillation; CHARGE: Cohorts for Heart and Aging Research in Genomic Epidemiology.

Figure 2 shows an age-adjusted analysis of high versus low physical activity levels. Participants who took ≥7500 steps daily had lower estimated CHARGE-AF risk scores than those of participants who took <7500 steps daily (mean 0.90% vs mean 1.3%; *P*<.001).

We observed significant interactions by sex and obesity; the association between the CHARGE-AF score and daily step count was stronger in men and participants with obesity. The CHARGE-AF risk score was 0.14% and 0.05% lower for every 1000 steps in men and women, respectively (interaction: *P*<.001). Similarly, the CHARGE-AF score was 0.10% and 0.03% lower for every 1000 steps in individuals with obesity and individuals without obesity, respectively (interaction: *P*<.001).

Participants’ self-reported physical activity levels, as determined by the PAI score, were not associated with the CHARGE-AF score.

Sensitivity analyses were conducted to investigate the association between daily step count and the CHARGE-AF score, using different device wear thresholds. The watch wear cutoff was increased to ≥60 days and ≥90 days from the cutoff of ≥30 days used in primary analysis. In a separate analysis, the wear time threshold was increased to 10 hours from the threshold of 5 hours used in the primary model. The inverse association between daily step count and the CHARGE-AF risk score remained significant in these subgroups (Tables S1 and S2 in Multimedia Appendix 1).

**Table 2:** Association between average daily step count and the predicted 5-year atrial fibrillation (AF) risk^a^.

	Change in AF risk score per 1000 steps (%), β^b^ (SE)	*P* value
All participants	−0.08 (0.01)	<.001
Men	−0.14 (0.02)	<.001
Women	−0.05 (0.01)	.001
No obesity	−0.03 (0.01)	<.001
Obesity^c^	−0.10 (0.03)	<.001

^a^The model was adjusted for age, sex (for the model including all participants), and wear time.

^b^β represents the change in 5-year AF risk for every 1000-step increase in daily step count.

^b^Obesity was defined as a BMI of ≥30 kg/m^2^.

## Discussion

### Main Findings

In our observational cross-sectional analysis of eFHS participants, the 5-year AF risk, as predicted by the CHARGE-AF score, was low (0.5% in women and 1% in men), and the score was 0.08% lower for every 1000 steps in a model adjusted for age, sex, and wear time. Interactions were significant for sex (*P*<.001) and obesity (*P*<.001); in men and participants with obesity, the association between the CHARGE-AF score and daily step count was stronger. Finally, self-reported physical activity, as determined by the PAI score, was not associated with the CHARGE-AF score. A graphic abstract that presents our main findings is presented in Multimedia Appendix 2.

### Relation Between Physical Activity and AF

The overall benefits of increased physical activity have been well documented, including reductions in all-cause mortality, CVD-related mortality, and overall CVD risk [12-15,27,28]. The relation between physical activity and the risk of AF is more complex. Studies that examined incident AF and physical activity suggested an increased AF risk with vigorous physical activity but a protective benefit of low to moderate physical activity against AF [29-33]. These studies however relied on self-reported physical activity [29-33].

The association between future AF risk and objectively measured physical activity has been investigated in both short-term accelerometer use and the long-term use of implantable devices (≥25 months). AF risk was either estimated with the CHARGE-AF score or calculated after monitoring for incident AF [17-20]. All of these methods detected an association between lower physical activity levels and a higher risk of AF, but these studies have limitations. In studies that used research-grade accelerometers, which are not commercially available, the device wear time was ≤7 days, and it may not have reflected an individual’s habitual, long-term physical activity lifestyle [17,18]. Additionally, these studies categorized physical activity into levels, such as “light,” “moderate,” or “vigorous.” This methodology aligns with the recommendations from public health organizations, which currently recommend ≥150 minutes of moderate physical activity per week or ≥75 minutes of vigorous physical activity per week. Although the use of step counts may result in the overestimation or underestimation of intentional physical activity, step count targets may be more understandable to and practical for the general population than the abstract concepts of moderate and vigorous levels of physical activity. Additionally, evidence is evolving to suggest that ≥7000 daily steps in adults may be equivalent to the current public health recommendation of ≥150 minutes of moderate physical activity per week or ≥75 minutes of vigorous physical activity per week [34]. Studies that use invasive devices, such as implantable loop recorders or implantable cardioverter-defibrillators, are less applicable to the general population, and such devices are not regularly used [19,20]. Our study may offer a more accurate window into a participant’s overall physical activity lifestyle and routine. As such, monitoring daily step count for a longer period of time with a commercially available noninvasive device may provide a more real-life reflection of the relation between physical activity and the estimated risk of AF. Additionally, our use of daily step count instead of a physical activity level (eg, moderate to vigorous physical activity) may provide a clearer and more practical target for AF risk reduction in the future.

Our study also demonstrated that sex and obesity may modify the association between AF risk and daily step count, as a stronger association between AF risk and daily step count was noted in men and participants with obesity. The independently increased risk of AF in men and individuals with obesity may have contributed to the stronger association that we observed between step count and the CHARGE-AF score [35].

Finally, our study did not find evidence of an association between self-reported physical activity and predicted AF risk. This lack of association may be related to the inconsistent validity of self-reported physical activity [36,37]. Our findings are consistent with the lack of a significant association between self-reported physical activity and the predicted risk of CVD [15].

The biological mechanisms behind the inverse association between step count, as an objective measure of physical activity, and estimated AF risk may be embedded within the shared risk factors between CVD and AF. On a cellular level, increased step counts have been associated with lower chronic inflammation and cardiac biomarkers, such as lipoproteins, white blood cell count, troponin, and N-terminal pro–brain natriuretic peptide [38-40]. As such, physical activity has been associated with lower atherosclerosis burden, as evidenced by decreased carotid artery plaque, thickness, and stiffness, as well as lower coronary artery calcification scores [41-43]. Finally, higher step counts and physical activity levels have also been associated with lower blood pressure and lower rates of obesity, coronary heart disease, and heart failure, of which all are major risk factors for AF [27,44,45].

### Study Limitations

Our study has several limitations. First, this study is limited by its observational and cross-sectional design, precluding the ability to establish causality, establish temporality, or rule out residual confounding. Additionally, the cross-sectional design precludes prospective follow-ups for the occurrence of incident AF. As such, the use of the estimated CHARGE-AF risk (as opposed to incident AF) as the primary outcome limits interpretations of clinical significance. However, CHARGE-AF risk has been extensively validated in large data sets with good predictive performance for incident AF [46-48]. A second limitation is the absence of heart rate as a variable in our regression model; heart rate data were unavailable to be contemporarily correlated with step count at the time of analysis. The lack of concurrent heart rate data may limit the inference of the association between physical activity and AF risk, but it does not affect the association between objectively measured step count and estimated AF risk. A third limitation is that the majority of participants in this study (n=838/923 (90.8%)) were White, middle-aged or older (age: mean 52 years) who were presumably living in Massachusetts. Additionally, compared to the FHS participants who did not enroll in the eFHS, the participants in the eFHS were younger, had higher levels of education, and had fewer CVD risk factors [24]. Further, the accuracy of wearable devices among individuals with darker skin tones is unclear [49,50]. Given these limitations, the applicability of this study to the general population is limited. Additionally, we anticipate participation and observation bias due to the language and smartphone eligibility requirements, as well as inaccuracies in measures derived from wearable devices, as previously reported [51]. The inference of physical activity level by using step counts may result in the overestimation and underestimation of vigorous physical activity.

### Conclusions

Increasing daily steps can be a practical, lifestyle-modifying method for reducing an individual’s AF risk. Future studies could investigate a dose-dependent relation between step count and AF risk and examine this relation in more ethnically diverse, racially diverse, and age-diverse populations. Given the emerging relation between AF risk, objectively measured physical activity, and daily step count, assessing commercially available wearable devices for AF preventative risk reduction merits further investigation.

## Appendix

Multimedia Appendix 1 Supplementary figure and tables.

Multimedia Appendix 2 Graphic abstract.

## Abbreviations

AF: atrial fibrillation
CHARGE: Cohorts for Heart and Aging Research in Genomic Epidemiology
CVD: cardiovascular disease
eFHS: electronic Framingham Heart Study
FHS: Framingham Heart Study
PAI: physical activity index
