# Supplementary material for: Step Count, Self-reported Physical Activity, and Predicted 5-Year Risk of Atrial Fibrillation: Cross-sectional Analysis
Source: J Med Internet Res. 2023 Mar 6;25:e43123. doi: 10.2196/43123 (PMC10028513; doi:10.2196/43123)
Supplement: Multimedia Appendix 1 [file jmir_v25i1e43123_app1.pdf]

This is a Multimedia Appendix to a full manuscript published in the J Med Internet Res. For full copyright and citation information see http://dx.doi.org/10.2196/jmir.xxxx

Figure S1: Flow Diagram of Participant Enrollment

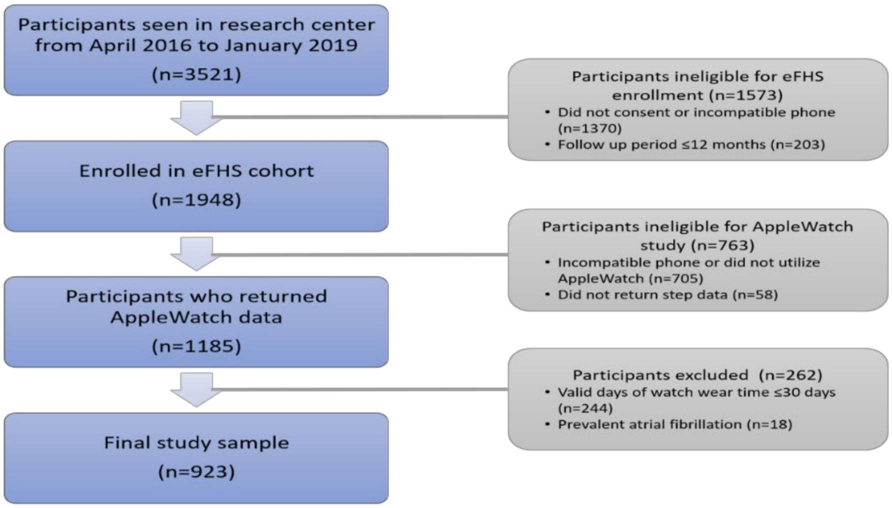

Table S1. Association of the average daily step count with 5-year predicted AF risk with different thresholds for valid days

	Change in AF risk score per 1000 steps [β† (SE)]	*P* value
≥60 valid days threshold‡	-0.07% (0.01)	<0.001
≥90 valid days threshold	-0.07% (0.02)	<0.001

* The model was adjusted for age, sex (for the model including all participants), and wear time.
† β represents the change in 5-year AF risk for every 1,000 steps increase daily.
‡ Valid days were defined as the days with ≥5 hours of watch wear time

Table S2. Association of the average daily step count with 5-year predicted AF risk with 10 hours per day wear time threshold for valid days

	Change in AF risk score per 1000 steps [β† (SE)]	*P* value
All participants	-0.07% (0.01)	<0.001
Men	-0.12% (0.02)	<0.001
Women	-0.05% (0.01)	0.001

* The model was adjusted for age, sex (for the model including all participants), and wear time.
† β represents the change in 5-year AF risk for every 1,000 steps increase daily.
