# Supplementary material for: Step Count, Self-reported Physical Activity, and Predicted 5-Year Risk of Atrial Fibrillation: Cross-sectional Analysis
Source: J Med Internet Res. 2023 Mar 6;25:e43123. doi: 10.2196/43123 (PMC10028513; doi:10.2196/43123)
Supplement: Multimedia Appendix 2 [file jmir_v25i1e43123_app2.pdf]

Graphic Abstract:

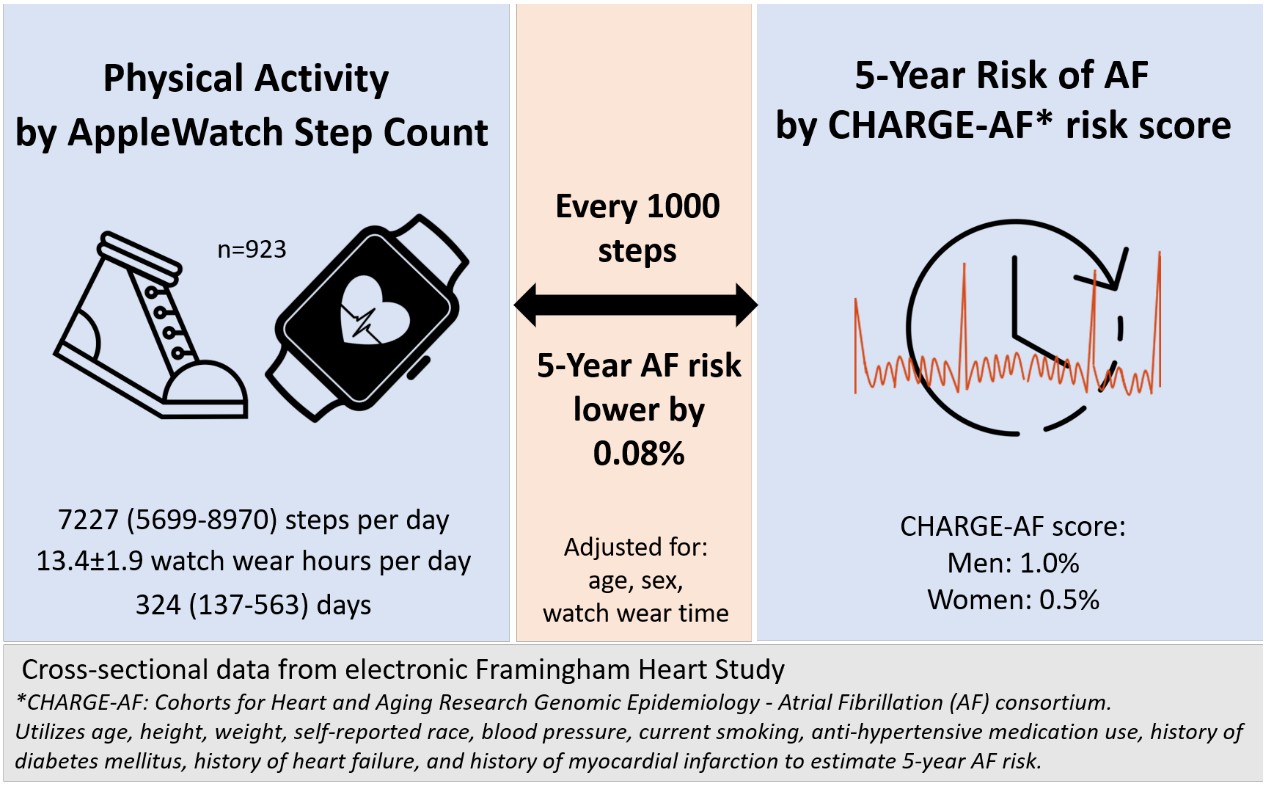

Predicted risk of atrial fibrillation was inversely associated with daily step count, such that every 1000 steps were associated with 0.08% lower five-year risk of atrial fibrillation.
